# Do parental attachment and prosocial behavior moderate the impairment from depression symptoms in adolescents who seek mental health care?

**DOI:** 10.1186/s13034-023-00680-1

**Published:** 2023-11-28

**Authors:** Marsida Hysaj, Mathilde R. Crone, Jessica C. Kiefte-de Jong, Robert R.J.M. Vermeiren

**Affiliations:** 1 Department of Child and Adolescent Psychiatry, Leiden University Medical Center, Leiden, The Netherlands; 2 Department of Public Health and Primary Care / LUMC-Campus The Hague, Leiden University Medical Centre, The Hague, The Netherlands

**Keywords:** Depressive symptoms, Parental attachment, Prosocial Behavior, Impairment from symptoms, Strength-based approach, Clinical setting

## Abstract

We investigated parental attachment and prosocial behavior as social protective indicators in adolescents (age 11–17) with symptoms of depression in a clinical setting. Specifically, we tested the moderating effect of these factors on the relation between symptoms of depression and their impairment on daily life. The Development and Well-Being Assessment, as completed by children, mothers, and fathers, was used, and hierarchical multiple regression analyses were conducted for these three perspectives. From the adolescents’ reports, we only found a significant effect of symptoms on impairment, indicating that a higher number of symptoms were related to higher impairment. For the mothers and fathers, a higher score on the adolescents’ prosocial behavior was related to a lower impairment from depression symptoms on the daily life of the adolescent and the family. Only for the mothers did a higher score on prosocial behavior buffer the effect of symptoms on impairment, while a higher parental attachment score was associated with a lower impairment. Further, when examining maternal and paternal attachment separately, only the mothers reported less impairment when perceiving that the adolescent was attached to the father. Paternal attachment even buffered the effect of symptoms on impairment. To conclude, our results indicate that social protective factors, from the parent’s perspective, are likely to have a beneficial effect in clinical practice and should be taken into account when examining impairment scores. Future studies should investigate whether additional protective indicators from the adolescents’ perspective, such as quality of parental attachment or family climate, may have a positive impact on their daily functioning.

## Introduction

Research has shown that social protective factors, in particular social factors related to the family environment [1–4] and the child’s social competencies [5], can help against the development of psychopathology and are likely to buffer the negative effects of mental health problems on daily functioning [6–10]. Even though the beneficial effects of these factors on mental health have been shown, this research has been mostly conducted on community samples, and little is known about their influence in a clinical setting. However, studying these protective factors in a clinical setting is important, as they may explain how children and adolescents with a similar severity of symptoms vary in their functioning [11]. In our study, we focus on the protective role of parental attachment as an indicator of close family environment and prosocial behavior as an indicator of social competence in adolescents who seek mental health care and display clinical symptoms of depression. Here, we will investigate whether and how the impairment from symptoms may be mitigated by these social protective indicators.

Impairment has been defined as a disability in everyday functioning due to symptoms [12] and has been mainly observed across four domains: family life, friendships, classroom learning, and leisure activities [13, 14]. Children and adolescents who suffer from depression are likely to be impaired in all of these areas [15, 16]. For example, cross-sectional and longitudinal studies have shown that impairments due to depression symptoms have interfered with how a child interacts with the family (through withdrawal or discord) [17], may develop lower quality peer relations [18] and shows lower school performance, such as lower grades or an inability to cope with school demands [19]. Further, having more symptoms may result in higher impairment. Moreover, impairment increases with age, and girls seem to be impaired twice as much as boys in adolescence [15, 20].

Therefore, understanding which factors may influence the relation between symptoms and impairment is highly important for clinical interventions. One of the factors that may explain differences in functioning in adolescents with similar symptoms may be the presence of protective factors. By focusing on such protective factors, this study draws on a strength-based approach [21]. Different from the traditional-risk approach, which places emphasis on reducing risks and where a protective factor is often identified as the absence of risk, in the strength-based approach, a protective factor is distinct from the risk factor, not reducing the risk itself but instead moderating the negative effects of those risks. These protective factors are also called buffers, as, in many situations, it is not feasible to reduce the risk factor [7, 21]. For example, in our study, the presence of clinical symptoms may not be eliminated, but their impairment on the adolescents’ daily life may well be mitigated through social buffering factors, such as feeling attached to the caregiver and prosocial behavior.

Evidence for the protective role of parental attachment comes from literature that indicates being in a protective family environment fosters secure attachment, which has shown to promote the development of social competence/skills, help children cope effectively with distress, and protect them against the development of psychopathology [5, 22, 23]. For example, secure attachment has been associated with less severe depression in adolescents [24]. Further, as demonstrated by a recent meta-analysis, being securely attached to both parents resulted in fewer internalizing behaviors in children than being securely attached to one parent [25]. Insecure attachment, on the other hand, has been theorized to play an important role in the development of depression symptoms and a clinical condition [23, 24, 26]. This has also been confirmed by recent studies, where insecure attachment was found to predict depression symptoms [22, 26, 27]. So far, we have observed that attachment has a positive influence on the severity of symptoms, but it is unclear how it actually influences impairment (i.e., the everyday suffering from the symptoms). As there is some evidence that secure attachment (to both parents) promotes better coping and a reduction in the severity of symptoms, secure attachment to caregivers may also lead to less impairment [22, 25].

Prosocial behavior represents one domain of social competence, also called others-oriented competence. Prosocial behavior is manifested by the voluntary action to share, help, and cooperate with others [5, 28]. Research has demonstrated that prosocial behavior has positive effects on mental health and other domains in children’s lives, such as academic achievement and the quality of peer relations. In both community and clinical samples prosocial behavior has been found to be negatively and positively related to symptoms of depression [18, 19]. Parents usually report fewer depressive symptoms in their child when scoring higher regarding the child’s prosocial behavior [28–30]. In children and adolescents, both positive and negative associations have been reported with prosocial behavior [31, 32]. A recent meta-analysis using non-clinical data showed that more prosocial behavior was weakly but significantly related to a decreased risk of depression [20]. However, the authors could not explain these effects based on the available studies. As they reported, the possible mechanisms that may explain the relation between prosocial behavior and depression symptoms have not previously been studied [30]. More research is needed to elucidate the relation between social competences, such as prosocial behavior and depression symptoms in particular, in a clinical sample that can be characterized by an elevated severity of symptoms. Furthermore, little is known about how prosocial behavior may specifically affect the daily functioning of adolescents with depressive symptoms in a clinical setting. It has, however, been proposed that assessing social competencies is important as they may provide information about functioning beyond the present risk factors [11]. Therefore, studying how prosocial behavior may influence impairment in adolescents with a similar severity of symptoms may provide us with valuable information about everyday functioning beyond the symptoms, offering a more complete picture of the adolescents’ situation.

In addition to studying parental attachment and prosocial behavior separately, there may be a combined effect of these factors on the impairment from symptoms, as suggested by a recent study, which found that secure attachment attenuated the negative association between symptoms and prosocial behavior, indicating that the securely attached children in their study showed more prosocial behavior despite the severity of their symptoms [33]. Support for testing this effect comes also from the positive developmental cascade model, which states that a positive factor will initiate a positive cascade of events and protect against negative outcomes [5, 34, 35]. For instance, strong attachment to a caregiver may foster prosocial behavior, and this association may further influence depression’s impairment on daily life.

Next to the adolescents’ perspective on social protective factors, we also will take into account the mothers’ and fathers’ perspectives. So far, little is known about the effect of prosocial behavior and parental attachment from a multi-informant perspective in a clinical setting. Different informants (child, father, and mother) often give a unique and valuable contribution to the needs of the child, resulting in different perceptions and awareness of mental health problems [36–38]. For example, as has been proposed in the literature, parents may be better at perceiving the more overt behavior of their child, while the child is more likely to also report less overt and observable symptoms, such as their feelings [11, 15, 16, 38].

As of yet, studies that measured agreement between informants on depression diagnosis/symptom severity of adolescents in a clinical setting have reported a moderate to high agreement between informants who see the child in a similar context (e.g., mother and father) and a low agreement between adolescents and parents [39–42]. Adolescents also report more symptoms than parents [15, 43, 44], and there is some evidence, albeit inconsistent [43], of adolescents reporting a higher rate of impairment due to their symptoms compared to parent reports [15, 16, 38]. Similar results on agreement between mother and father and between parents and adolescents have also been reported by the few studies measuring social competence/prosocial behavior in both non-clinical and clinical settings [11, 29, 45, 46]. Further, there is some preliminary evidence that adolescents rate their prosocial behavior higher than their parents [29, 47] and that they may score high on prosocial behavior even when they report high depression symptoms [32]. Parents, on the other hand, when reporting more prosocial behavior of their child, scored lower on depression symptoms [28, 29]. These findings suggest that prosocial behavior may play a more important role in predicting impairment according to the parents than according to the adolescents, meaning that, when parents report lower prosocial behavior of their child, they score higher on impairment in daily life and vice versa. There is no study that has measured agreements on attachment between the adolescent, mother, and father perspectives. From the literature, however, we know that mothers are still more involved in childrearing tasks and spend, on average, more time with their children than fathers [25, 48] and that they tend to be more sensitive and responsive in soothing and comforting their child when distressed [25, 48–50]. Due to their time spent with the child and their sensitivity towards social interaction, mothers in particular may be more likely to report less impairment from the adolescent’s depressive symptoms when they perceive good attachment.

At present, it is unclear to what extent an adolescent’s feeling attached to the caregiver and prosocial behavior moderate the relation between symptoms of depression and their impairment on daily life in adolescents who seek mental health care. This is what we aim to investigate in our study, using a strength-based approach. We expect that adolescents who are better attached to their caregivers will show less impairment from their symptoms due to the protective effect of being better attached. We also expect a lower degree of impairment from symptoms for adolescents who score higher on prosocial behavior. Furthermore, we expect that adolescents who are better attached to their caregivers and who show more prosocial behavior will particularly benefit from this combination, leading to a reduced impairment from symptoms. Finally, by investigating these factors with different informants (child, mother, and father), we hope to better understand the role of social protective factors from different perspectives. If our findings support our hypotheses, this may be useful information for clinicians to prioritize interventions that focus on the positive role of these indicators and, in general, on the positive role of social environment.

## Methods

### Participants and procedure

This study used data from adolescents who were admitted to the center for Child and Adolescent Psychiatry (LUMC Curium) for various psychiatric disorders. The medical ethics review board of the Leiden University Medical Center judged the overall study and stated that the research is not subject to the Medical Research Involving Human Subject Act (non-WMO approval number: G21.174). This study has been further approved by the scientific committee of LUMC Curium. To examine the relation between symptoms and impairment we used the Development and Well-Being Assessment (DAWBA), which is employed to generate DSM-V psychiatric diagnoses [51]. The DAWBA is administered standardly to all children and adolescents referred to LUMC Curium. For the purpose of this study, we only made use of the data at referral. At referral, parents, adolescents (from the age of 11) and teachers are required to complete the DAWBA as a first step in the diagnostic process [51]. We included DAWBA’s that were administered between 2015 and 2020 and those in which, next to a self-rating of the adolescent, at least one parent rating should was present, reducing the total number to 1203 DAWBA’s. From these, a random sample of 750 participants was chosen. In total, 750 adolescents, 636 mothers, and 540 fathers completed the DAWBA. Finally, only participants with data on impairment scores for depression symptoms were included (i.e., participants with core symptoms of depression). For the adolescents, 341 had completed the impairment questions, for the mothers 261, and for the fathers 193. Of these 341 children, 191 mothers, and 131 fathers reported the symptoms and the impairment scores^1^.

We conducted extra analyses to assess whether participants included in our analyses differed from those who were not included in the final sample. We found that parents in our final sample showed significant differences from those with no data on impairment. No data on impairment meant that according to the parents adolescents didn’t suffer from depression symptoms. Specifically, as rated by both parents, adolescents who were included in our study exhibited on average lower prosocial behavior than those not included. Further, the included adolescents were on average older and the proportion of girls was higher. Moreover, the likelihood of experiencing anxiety next to depression symptoms was significantly higher in the included group. In the adolescent data, we found comparable results for age, gender, and likelihood of anxiety disorders as reported by the parents. The adolescents included in our study showed no significant differences in prosocial behavior compared to those who were not included.

### Measures

#### The DAWBA and DSM-V depression

The DAWBA is a widely used computerized diagnostic interview that includes structured questions and interviews about symptoms and impairment per DSM qualification in 2–17 year-old children [12, 51] and has shown good predictive validity for emotional disorders [52]. The questions are closely related to the DSM-V diagnostic criteria on current problems. When looking at the DSM-V, five or more symptoms are required for a depression diagnosis [53], one of which should be depressed mood, irritability, or loss of interest- also referred to as core symptoms- with at least four other symptoms. When assessing depression via the DAWBA a participant needs to have answered “yes” for at least one core symptom in order for the participant to continue with questions on other symptoms and the impairment from depressive symptoms on daily life.

From the DAWBA, 11 symptoms are specifically related to depression: three core- and eight secondary symptoms. The following question is related to the core symptoms: “In the **last 4 weeks**, have there been times when you have been 1) very sad or 2) grumpy or 3) have lost interest? And did you feel this way almost **every day** and for **most of the day?**” The following question is an example of a secondary symptom: “Did you lack energy or seem tired all the time?” Each item is rated on a unidimensional scale with possible answers of 0 “no” or 1 “yes”. The symptoms were summed to a score of 1 to 11.

#### Parental attachment

We used the “attachment scale” in the DAWBA (parents’ and children’s perspective). This scale measures the number of key attachment figures to which children feel securely/strongly attached to. Items are rated on a two point scale with possible answers of 0 “no” and 1 “yes”. Here, we focused particularly on parental attachment. Adolescents were shown the following text: “Most young people are particularly attached to a few key adults, looking to them for security and comfort, and turning to them when upset or hurt. Are you particularly attached to the following adults: Mother (biological or adoptive), Father (biological or adoptive), Another mother figure (stepmother, foster mother, father’s partner), Another father figure (stepfather, foster father, mother’s partner)?” We aggregated the items of mother + another mother figure into one “Mother” variable and father + another father figure into one “Father” variable^2^. The total score of parental attachment could be 0 “no attachment to parental figures,” 1 “attached to one of the parental figures,” or 2 “attached to both parental figures.” Next to parental attachment, we also assessed maternal and paternal attachment separately.

#### Prosocial behavior scale

In our study, we used the degree of prosocial behavior as measured by the Strength and Difficulties Questionnaire (SDQ) [12] in the DAWBA for the parent- and self-reports on depression. The SDQ is a brief screening instrument for mental health problems and, in addition to difficulties (emotional or behavioral), also contains strength items (prosocial behavior). The instrument can be completed by children, parents, and teachers. The prosocial subscale was shown to have a good internal consistency for both parent- and self-reports (Cronbach’s alpha = 0.75) in a sample of adolescent clinical outpatients [54]. In our study, the reported Cronbach’s Alpha for the adolescents was lower than that reported in the literature (Cronbach’s alpha = 0.64), while it was comparable to the literature reports for the parents (Cronbach’s alpha = 0.73). Prosocial behavior is manifested when persons help, share, and/or cooperate with others. This scale consists of five items rated on a three-point scale, with possible answers of: 0 “Not true,” 1 “Somewhat true,” and 2 “Certainly true.” An example of such an item from the adolescents’ perspective is: “I am kind to younger children.” The score can range from 0 to 10 if all items are completed. Higher scores correspond to more prosocial behavior.

#### Impairment

We used the DAWBA impairment scores for the parent- and self-reports on depression to indicate the adolescent’s level of distress and functional impairment from the symptoms. In our study, a Cronbach’s Alpha of 0.76 and 0.67 was found for the impairment scale for the adolescents and the parents, respectively. In the DAWBA, there are six questions about impairment per disorder: one item about distress; four items on social impairment in (a) family life, (b) friendships, (c) learning, and (d) leisure activities; and one item about burden [51]. An example of such an item from the adolescents’ perspective is: “Has your sadness, irritability, or loss of interest interfered with how well you get along with the rest of the family?” Each item is rated on a three-point scale with answer categories ranging from: 0 “Not at all” and 3 “A great deal.” Importantly, impairment questions are not related to the impairment associated with specific depression symptoms; rather, they concern the overall impairment from depression symptoms on daily functioning. We used a sum score to calculate impairment.

#### Control variables

As low Social Economic Status (SES) and migration status have been considered risk factors for depression in adolescents, we controlled for the influence of these factors in our study [22].

#### Social economic status

The Social Economic Status (SES) of the participants was assessed using neighborhood status scores, which combined the average income with the level of education and the proportion of unemployed individuals in a neighborhood [55]. The average SES score in the Netherlands in 2010 was 0.17 (-7.25–3.19).

#### Ethnicity

For ethnicity, the recent definition of Statistics Netherlands was used [56]. Based on this definition, five categories were created: (1) If the child was born in the Netherlands and the parents are Dutch, we classified this as having a Dutch origin. (2) Children that are born abroad are referred to as migrants. The rest of the categories were children who are born in the Netherlands and have at least one foreign-born parent from (3) Europe, (4) classical migrants groups in the Netherlands (such as Morocco, Turkey, Suriname, Indonesia, and Netherlands Antilles) or (5) outside Europe. These are called children of migrants. To control for the influence of ethnicity in our statistical model, we created four dummy variables of ethnicity using the Dutch-origin group as the reference group.

#### Other disorders

To offer a characterization of other possible disorders in our sample, the DAWBA bands on five other disorders were used. DAWBA bands are created based on information about symptoms and impairment from symptoms from multi-informants on all DSM-IV disorders [22, 57]. Then, with the help of an algorithm, this information is aggregated to determine the likelihood of having a disorder. The DAWBA bands offers six possible answer categories (0.1%, 0.5%, 3%, 15%, 50% or higher and greater than 70% probability) of having a disorder. Based on the literature, scores of 50% or higher have shown to predict a disorder well [52]. Therefore, in our study we used this as a criterium for the likelihood of having a disorder. To reduce the number of variables, we clustered anxiety disorders into one group and created a dummy variable of “Having an anxiety disorder” based on answers: 1 “yes” and 0 “no.” Further, based on the frequencies with which disorders occurred in our sample, we also included four other disorders, namely the eating disorders, Attention Deficit Hyperactivity Disorder (ADHD), Oppositional Defiant Disorder (ODD), and Conduct Disorder (CD). Similarly, for these disorders, we created dummy variables.

### Analytical approach

First, to assess the association of impairment from symptoms with predicting variables and possible explanatory variables such as age, gender, SES, ethnicity, and other disorders, correlation and regression analyses were performed. Second, to test our main hypothesis (i.e., the buffering effect of social protective factors on impairment), hierarchical multiple regressions were used. We included separate models, for mothers, fathers, and adolescents. In all our models, (adolescent, mother, and father data) we first included the covariates (i.e., age, gender, SES, ethnicity and other disorders [Step 0]), followed by the predictors (i.e., depression symptoms, parental attachment, prosocial behavior, [Step 1]), and then, the interaction of parental attachment with symptoms on impairment, the interaction of prosocial behavior with symptoms and, lastly, adding the combination of prosocial behavior and parental attachment on impairment (Step 2). Importantly, this combined effect was not used as a moderator between depression symptoms and impairment, but as a combined main effect on impairment. Moreover, we reconducted all our analyses using the maternal and paternal attachment variables separately and also in a group where adolescents’ data was linked to their parents. We used two tailed-test in all analyses and *p* < 0.05 value as an indicator of statistical significance. All the analysis were conducted in SPSS version 29.

## Results

### Participants’ characteristics

The means and standard deviations of our main and control variables are shown in Table 1. The average age of the adolescents was between 14 and 15 years old, about 70% were girls and the majority were of Dutch origin. Based on the descriptive statistics, the adolescents reported on average a higher number of symptoms and scored higher on prosocial behavior than their parents. The parents, on the other hand, reported a higher score on impairment. Further, 96.5% of the mothers perceived that their child was attached to them, compared to 80% of the fathers. Both parents and adolescents reported the comorbidity with anxiety symptoms.

Furthermore, low to moderate correlations were found when comparing the scores of impairment, symptoms, prosocial behaviour and attachment between parents, and between parents and adolescents. Correlations varied between − 0.02 and 0.29 for adolescents and their fathers, 0.29 to 0.58 for adolescents and their mothers, and − 0.04 to 0.60 for mothers and fathers, indicating that they have partly different perspectives on impairment, symptoms, prosocial behaviour and attachment. The highest correlation between mothers and fathers were found for adolescents’ prosocial behaviour (r = 0.60, *p* < 0.01) and between mothers and adolescents for paternal attachment (r = 0.58, *p* < 0.01).

**Table 1:** Means (M) and standard deviations (SD) of our main and control variables

	Adolescents (N = 341)		Mother (N = 261)		Father (N = 193)	
Age (M, SD)	14.45	1.67	14.33	1.67	14.41	1.63
Gender female (%)	73%		67%		73%	
Socioeconomic status	0.60	0.74	0.56	0.84	0.60	0.88
Ethnicity (%)	85.6%		86.6%		85%	
Anxiety Bands (%)	71.5%		73.5%		69.3%	
Eating Disorder Bands (%)	13%		11.5%		13%	
ADHD Bands (%)	10.6%		16.5%		11.4%	
ODD Bands (%)	25.4%		33%		26.9%	
CD Bands (%)	11.8%		15.3%		11.9%	
Symptoms of depression^a^ (M, SD)	8.15	2.09	7.47	2.12	6.79	2.21
Parental attachment^b^ (M, SD)	1.62	0.63	1.75	0.48	1.73	0.54
Maternal attachment (%)	87.3%		96.5%	93%		
Paternal attachment (%)	74.4%		78.8%	80%		
Prosocial behavior^c^ (M, SD)	7.71	1.87	6.62	2.27	6.32	2.40
Impairment symptoms^d^ (M, SD)	10.31	4.06	11.89	3.51	11.59	3.24

^a^DAWBA depression scale range: 1–11

^b^DAWBA parental attachment scale range: 0–2

^c^SDQ prosocial scale range: 0–10

^d^DAWBA impairment scale range: 0–18

### Associations of predicting variables on the impairment from symptoms

We calculated the correlations and 95% confidence intervals of predicting variables on impairment, as shown in Table 2. When testing the univariate effects of these variables, only from the adolescents’ perspective did we observe a significant correlation between age and gender on the impairment from the symptoms. This implies that, according to the adolescents, the impairment from the symptoms seems to be higher as the children age, and girls seem to report higher impairment scores. Overall, we found a significant correlation between symptoms and impairment, which indicates that a higher number of symptoms is associated with higher impairment scores. Only for the parents, we found a significant association between prosocial behavior and impairment in their children. Further, only mothers reported a negative association between parental attachment and impairment, indicating that more prosocial behavior and stronger parental attachment is associated with a lower impairment. Additional inspection of the data showed a negative association for paternal attachment and impairment in the model with mother ratings, while the correlation between their own attachment and impairment was non-significant. Furthermore, both adolescents and parents reported significant associations with anxiety bands, indicating the comorbidity of depression with anxiety symptoms. Finally, only for parents a significant correlation between CD and ODD bands and impairment was reported.

**Table 2:** Associations of predicting and control variables on impairment

	Adolescent reported impairment		Mother reported impairment		Father reported impairment	
	***r***	(95%CI)	***r***	(95%CI)	***r***	(95%CI)
Age	0.12*	**(0.04–0.56)**	0.05	(-0.16-0.36)	-0.09	(-0.46-0.11)
Gender						
*Girls (Reference)*	0		0			0
*Boys*	-0.15**	**(-2.45–0.39)**	-0.012	(-1.00-0.82)	-0.03	(-0.82-1.26)
Socioeconomic status	0	(-0.59 - -0.57)	-0.063	(-0.78-0.25)	-0.03	(-0.64-0.39)
Ethnicity						
*Dutch (Reference)*	0		0		0	
*Europe*^*a*^	0.03	(-1.72-2.83)	0.11	(-0.16-4.51)	0.11	(-0.56-4.04)
*Classic migrant group*^*a*^	0.02	(-2.11-3.32)	-0.1	(-5.28-0.41)	-0.05	(-3.61-1.67)
*Outside Europe*^*a*^	-0.04	(-3.05-1.34)	-0.03	(-2.60-1.64)	-0.12	(-4.31-0.29)
*Migrants*	0.03	(-1.72-2.83)	-0.054	(-3.38-1.29)	0	(-2.51-2.39)
Anxiety Bands	0.23**	**(0.13–0.33)**	.25**	**(0.13–0.36)**	0.22**	**(0.08–0.35)**
Eating Disorder Bands	0.17**	**(0.07–0.28)**	0.06	(-0.06-0.18)	-0.03	(-0.17-0.11)
ADHD Bands	0.04	(-0.07-0.14)	0.07	(-0.05-0.19)	-0.01	(-0.15-0.13)
ODD Bands	0.06	(-0.05-0.17)	0.18**	**(0.06–0.30)**	0.15*	**(0.00-0.29)**
CD Bands	0.11*	**(0.01–0.22)**	0.21**	**(0.09–0.33)**	0.17*	**(0.03–0.30)**
Symptoms	0.55**	**(0.89–1.24)**	**0.41** ^**^	**(0.50–0.87)**	**0.35** ^**^	**(0.32–0.72)**
of depression						
Parental attachment	0	(-0.92-0.49)	**-0.17** ^**^	**(-2.08- -0.31)**	0	(-0.87-0.89)
Maternal attachment	0.05	(-0.06-0.16)	-0.04	(-.016-0.09)	0.02	(-0.12-0.17)
Paternal attachment	-0.08	(-0.19-0.03)	-0.18**	**(-0.30- -0.06)**	-0.01	(-0.16-0.13)
Prosocial behavior	-0.04	(0.32 − 0.15)	**-0.24** ^**^	**(-0.55- -0.18)**	**-0.21** ^**^	**(-0.47- -0.09)**
Symptom*Attachment	0.01	(-0.31-0.39)	0.07	(-0.17-0.70)	0.04	(-0.31-0.54)
Symptom*Prosocial	0.02	(-0.09 -0.13)	0.12	(< 0.01–0.19)	-0.01	(-0.09-0.81)
Attachment*Prosocial	0.05	(-0.18-0.50)	-0.08	(-0.58–0.12)	0.08	(-0.16-0.50)

*P < 0.05, **P < 0.01, ^*a*^=Children of migrants

### Predicting impairment from the adolescents’ perspective

After controlling for age, gender, SES, ethnicity and other disorders, only depression symptoms were found to significantly predict impairment. Further, we found no main effects of parental attachment or prosocial behavior on impairment (Table 3). These results demonstrated that adolescents with a higher number of symptoms experienced a higher level of impairment from these symptoms in their daily life. This was also the model that explained most of the variance (Step 1, model), with an R^2^ = 0.33. Moreover,, no interaction effects of parental attachment or prosocial behavior with symptoms on impairment were reported. In addition, we reconducted the same analyses using the maternal and paternal attachment variables separately, and the results remained the same. The only significant effect was the main effect of symptoms on impairment (β = . 47, ρ < 0.01).

**Table 3:** The effects of predicting variables on the impairment from symptoms (adolescents’ perspective, DAWBA)

	*Total R* ^*2*^	*R* ^*2*^_*incl.*_	*B*	*SE B*	*β*
Step 0:	0.14				
Age			0.29	0.13	0.12*
Anxiety Bands			2.01	0.48	0.24**
Eating Disorder Bands			2.01	0.64	0.17**
Step 1:	0.33	0.19**			
Symptoms of depression			0.88	0.01	0.47**†
Parental attachment			0.13	0.32	0.02
Prosocial behavior			− .07	0.11	− .03
Step 2:	0.34	0.01			
Symptoms*Parental attachment			− 0.13	0.15	− 0.04
Symptoms*Prosocial behavior	0.33		0.00	0.05	0.00
Parental attachment* Prosocial			0.17	0.15	0.06

B = unstandardized regression weight, *SE B* = standard error of B, * *p* < 0. 05, ** *P* < 0.01. The model was adjusted for age, gender, SES, ethnicity, and other disorders (i.e., Anxiety Bands, Eating Disorder Bands, ADHD Bands, ODD Bands and CD Bands). To save space, only covariates with a significant effect on impairment are presented in the table. All steps are incremental, variables of previous steps are included in the model but to save space are not presented in the table. R^2^ change effect size conventions: 0.01 = small, 0.06 = medium, 0.14 [58]

† Due to some missing values in parental attachment and SES, a total of 322 adolescents, 254 mothers, and 184 fathers were included in the main analysis.

### Predicting impairment from the mothers’ perspective

In the model with the mothers’ ratings (see Table 4), both parental attachment and prosocial behavior demonstrateda significant main effect on impairment (Step 1), even after controlling for depression symptoms and the covariates. These results suggest that, according to the mothers, when the adolescent shows high prosocial behavior and feels strongly attached to their caregivers, they observe a better functioning of the adolescent. Also, a significant interaction of prosocial behavior with symptoms was reported (Step 2), indicating that, according to the mothers, children with a similar number of symptoms but a higher score on prosocial behavior experienced less impairment from these symptoms compared to children with lower scores on prosocial behavior. We did not find a moderation effect of parental attachment with symptoms on impairment, while the combined effect of parental attachment and prosocial behavior on impairment was significant. Additionally, when reconducting these analyses for maternal and paternal attachment separately, the significant effect that we previously found in the mother sample between parental attachment and impairment was specifically attributed to their perceived father attachment, meaning that mothers perceived that, especially when they thought that adolescents were attached to their fathers, that this may result in less impairment (β = − 0.47, ρ < 0.01). Also, the interaction of paternal attachment with symptoms was significant (β = -0.15, ρ < 0.05). All the other results remained the same.

**Table 4:** The effects of the predicted variables on the impairment of symptoms (mothers’ perspective, DAWBA)

	*Total R* ^*2*^	*R* ^*2*^_*incl.*_	*B*	*SE B*	*β*
Step 0:	0.15				
Anxiety Bands			1.91	0.52	0.24**
CD Bands			1.66	0.67	0.17*
Step 1:	0.29	0.14**			
Symptoms of depression			0.55	0.10	0.34**
Parental attachment			-0.84	0.41	-0.12*
Prosocial behavior			-0.28	0.09	-0.18*
Step 2:	0.32	0.03*			
Symptoms*Parental attachment			0.23	0.20	0.07
Symptoms*Prosocial behavior			0.01	0.04	0.13*
Parental attachment* Prosocial			-0.37	0.17	-0.13*

B = unstandardized regression weight, *SE B* = standard error of B, * *p* < 0. 05, ** *P* < 0.01. The model was adjusted for age, gender, SES, ethnicity and other disorders (i.e. Anxiety Bands, Eating Disorder Bands, ADHD Bands, ODD Bands and CD Bands). To save space, only covariates with a significant effect on impairment are presented in the table

### Predicting impairment from the fathers’ perspective

As presented in Table 5, there was a main effect of prosocial behavior on impairment, showing that, the fathers just as mothers perceive high prosocial behavior of the child to be associated with a lower impairment of the symptoms. However, neither prosocial behavior nor parental attachment moderated the relation between symptoms and impairment. In addition, when reconducting the same analyses using maternal and paternal attachment variables separately, the results remained the same as in the model where parental attachment was included. The only significant effect were the main effect of symptoms (β = 0. 33, ρ < 0.01) and prosocial behavior (β = -0.24, ρ < 0.01) on impairment.

**Table 5:** The effects of predicting variables on the impairment of symptoms (fathers’ perspective, DAWBA)

	*Total R* ^*2*^	*R* ^*2*^_*incl.*_	*B*	*SE B*	*β*
Step 0:	0.13				
Anxiety Bands			1.89	0.54	0.27**
Step 1:	0.26	0.13**			
Symptoms of depression			0.50	0.11	0.34**
Parental attachment			0.91	0.44	0.15
Prosocial behavior			-0.31	0.01	-0.23**
Step 2:	0.27	0.01			
Symptoms*Parental attachment			0.12	0.21	0.06
Symptoms*Prosocial behavior			-0.05	0.05	-0.07
Parental attachment* Prosocial			0.00	0.18	0.00

B = unstandardized regression weight, *SE B* = standard error of B, * *p* < 0. 05, ** *P* < 0.01. The model was adjusted for age, gender, SES, ethnicity, and other disorders (i.e. Anxiety Bands, Eating Disorder Bands, ADHD Bands, ODD Bands and CD Bands). To save space, only covariates with a significant effect on impairment are presented in the table

## Discussion

We examined the moderating role of parental attachment and prosocial behavior on the relation between symptoms and impairment in a group of adolescents with depression symptoms in a clinical setting. A higher number of symptoms was significantly related to greater impairment from the perspective of the adolescent, mother, and father. Only for the mothers and fathers did we find that, if they reported more prosocial behavior of the adolescent, they reported less impairment from depression on the daily life of the adolescent and family. Only for the mothers was a higher score on parental attachment related to a lower score on impairment. Further, when examining maternal and paternal attachment separately, we found that, for the mothers, when they perceived the adolescent as being attached to their father, they reported less impairment from their symptoms and even a buffering effect of the symptoms on impairment. Finally, and only according to the mothers, a higher score on prosocial behavior buffered the effect of symptoms on impairment, and both greater prosocial behavior and parental attachment of the adolescent were associated with less impairment.

We investigated parental attachment as a social protective indicator based on literature in which secure attachment is linked to less severe depression [24] or attenuated symptoms of depression [3]. In addition, based on the strength-based approach we tested the moderating effect of parental attachment on impairment. Nevertheless, we did not find a buffering effect when using parental attachment. An explanation for this may be that the parental attachment scale was only able to distinguish whether the child felt securely attached to none, one, or both parents. However, it could not differentiate between attachment styles and to what extent the child felt securely attached to parents as the items were measured in a unidimensional way with answer categories of “no” and “yes.” Future research may include more specific measures on the quality of parental attachment.

Although we found a direct, significant effect of parental attachment on impairment, from the mother perspective only, by further examining mothers’ perceived maternal and paternal attachment separately, we observed that this effect was particularly attributed to the father attachment, meaning that when mothers perceived that adolescents were attached to their fathers, they reported less impairment. The association of paternal attachment with impairment even buffered the effects of the symptoms. This relation was not found for their own perceived attachment with the child. An explanation for these effects may be that nearly all mothers (96%) reported that their child was securely attached to them compared to 78% of the mothers reporting secure attachment to the fathers. This may suggest that the mother feels more supported when the father is also attached to the child and therefore may feel that the child is less burdened by thesymptoms. Furthermore, as mothers are more sensitive towards social interactions they seem more likely to report less impairment when they perceive good attachment with both parents. Finally, our findings are in line with studies that support the protective role of secure attachment against the development of depression [22, 25].

Further, we assessed the moderating role of prosocial behavior on the relation between symptoms and impairment based on literature stating that prosocial behavior has positive effects on mental health and protects against symptoms of depression [30, 35]. Only from the mothers’ perspective did we find a buffering effect, which is in favor of the strength-based approach, indicating that, despite of showing a similar severity of symptoms adolescents with greater prosocial behavior may suffer less from the effect of these symptoms compared to adolescents who score lower on prosocial behavior. As discussed in the previous paragraph, this finding may reflect that the mothers perceived greater benefits of social protective indicators on the functioning of the child compared to the fathers and children reports. Although the adolescents scored high on prosocial behavior, this was not associated with the number of symptoms nor did it seem to influence the relation between symptoms and impairment.

Adolescents also did not report a direct effect of prosocial behavior on impairment; while other studies based on the children’s ratings have shown significant effects, the obtained results were mixed [31]. Parents, on the other hand, reported that prosocial behavior has a negative effect on impairment, which suggests that more prosocial behavior may lead to less impairment from the child’s depression on the daily life of the child and family. Our results are in line with a recent meta-analysis that reported a weak significant negative effect between prosocial behavior and depression symptoms [30]. The different perceptions between the parents and children may be explained by their different perspectives, as also reported in our study when comparing scores on impairment, symptoms, prosocial behavior, and attachment. Studies show that, due to their less observable and private nature, adolescents may be a better source of information to report internalizing symptoms and how these symptoms may influence impairment [11, 38]. Yet, they may find it more difficult to consider how they relate to others [11]. Conversely, parents may be better able to observe behavioral expressions of prosocial behavior but are less able to detect the emotions or internal states of the child [38]. Moreover, parents observe the influence that prosocial behavior has on the family. When the child exhibits prosocial behavior, they may perceive the effects of the symptoms as less negative resulting in a lower impairment score.

Nonetheless, the reasoning behind adolescents being a better source of information for reporting internalizing symptoms and parents for reporting behavior does not explain why we found a negative relationship between prosocial behavior and impairment in the parents and not in the child. It may be that other processes in the child are at play, such as negative self-evaluation. Negative self-evaluation has been found to be a strong predictor of the severity of depressive symptoms, even when children with depression reported comparable scores in prosocial attributes as the community sample [59]. Similarly to our study, children scored high in prosocial attributes, but no association was found between these attributes and how they evaluated impairment on their daily life. These findings emphasize the need to better understand how prosocial behavior may influence impairment, as research has shown that social competencies may reduce the impairment of internalizing problems [11]. We recommend that future studies in a clinical setting use behavioral observations of prosocial behavior in addition to self-reports so that actual competencies are measured instead of only perceptions. Additionally, assessments of the motivation behind the prosocial behavior may be valuable to understand what drives adolescents to behave prosocially. For example, adolescents may show more prosocial behavior due to peer pressure or more altruistic reasons. These motivations could influence the effect of the behavior on the adolescent’s wellbeing.

In line with previous research on positive developmental cascades, we also investigated the combined effect of parental attachment and prosocial behavior on impairment [33]. Here, we found a significant combined effect only from the mothers’ perspective. As the literature on positive developmental cascades has proposed, proximal effects of secure attachment on the child’s functioning may beget further competencies that ultimately encourage the development of prosocial behavior [5]. This combined effect may further facilitate other processes “adaptive cascades,” such as better interaction of the child with the parents, family, and peers, protecting the child against negative mental health outcomes [5, 34, 35]. If we translate this model to our results, we noticed that mothers in particular found the combined association (i.e., feeling securely attached to both parents and exhibiting higher prosocial behavior) important. This effect may be explained in two ways: A child with depression symptoms who shows prosocial behavior may stay better attached to the parents, as a result of which less impairment may be experienced. The opposite may also be possible, in that a child with depression symptoms who is strongly attached to the parents may exhibit more prosocial behavior and therefore may be less burdened by the symptoms. However, why we found this effect only in the mothers and not in the fathers needs further investigation. Finally, as this study is cross-sectional and we did not test causal pathways the results should be interpreted cautiously and should be tested in longitudinal data.

Our study has a number of strengths: (1) We used data from a real clinical setting using validated instruments; (2) by including parental- and self-reports, a better representation of the context was offered for this group of adolescents in a clinical setting; (3) our variables included the protective role of parental attachment and prosocial behavior using a strength-based approach instead of solely focusing on problem areas; and (4) by controlling for individual factors such as gender, age, SES and ethnicity, we attempted to depict a complete picture of our population. This is in line with the recommendation of Memmott-Elison (2020) [30], which argued for the inclusion of demographics as it contributes to a better understanding of the social context of the child. Furthermore, by controlling for other disorders, a better characterization of our study population was offered. Finally, by finding the same results before and after controlling for other disorders, strengthened the effects of our findings.

There are two main limitations that consider attention: (1) As the data was not collected with our research question in mind, we could only include the two social protective factors that were available in our data set. Follow-up studies should include additional indicators, such as the quality of parental attachment, parental monitoring, family climate, quality of peer relationships, and participation in sports and social activities. (2) Our study is cross-sectional; therefore, no conclusions can be drawn about the causal relations of these social protective factors in a clinical setting. Future studies should integrate a longitudinal design and investigate how these effects may influence mental health outcomes of children over time.

### Clinical implications

As our study included only two social protective indicators it may be premature to draw strong conclusions for future clinical implications. However, what our results indicate is that including protective factors next to risk factors is important to gain a better understanding of children’s functioning and that these factors should be taken into consideration when developing and implementing clinical interventions. What became evident from our findings was the importance of paternal attachment in relation to impairment from the mother’s perspective. Therefore, when assessing impairment, attachment to both parents should be taken into account in the clinical practice. Furthermore, as already reported in previous research, our study showed that including parental reports alongside the adolescents’ reports may provide useful information about the behavior of the adolescent. As the adolescent may find it more difficult to assess how they relate to others, parents may give important information about their behavior and how that behavior may influence impairment. Therefore, including parental reports alongside adolescents reports may offer valuable information about assessing the adolescent’s functioning.

## Data Availability

We used existing data from patient files and at this moment there is no policy within the department about publishing these data. If the policy allows we will follow the guidelines and be as open as possible.

## Footnotes

1 Of the 341 children who reported impairment scores, 103 mothers (30%) reported no impairment, while data from 47 mothers (14%) was missing. For the fathers, 118 (34%) reported no impairment, while data from 92 fathers (27%) was missing.

2 In the father sample, fathers reported that 2.2% of the adolescents were attached to another father figure (i.e., step or foster parent), and 6.5% to another mother figure. In the mother sample, mothers reported that 2.4% of the adolescents were attached to another mother figure, and 10.6% to another father figure. In the adolescent sample, adolescents reported that 7.1% of them were attached to another mother figure, and 9% to another father figure.
